# Regional Differences Between Drug-Coated Balloons and Drug-Eluting Stents in *De Novo* Coronary Disease

**DOI:** 10.14740/cr2252

**Published:** 2026-08-31

**Authors:** Gulshan Man Singh Dangol, Quang Le, Sajana Maharjan, Nathalia Schettino Samad, Gianna Carolinne Graff Caletti, Mathews Rezende da Costa, Maria Eduarda Cavalcanti Souza, Kalgi Modi

**Affiliations:** a Department of Medicine, HSHS St. John’s Hospital, Springfield, IL 62701, USA; b University of Kansas Medical Center, Kansas City, KS 66103, USA; c New York University CAS, New York, NY 10003, USA; d Universidade de Passo Fundo, Passo Fundo, RS, Brazil; e Amazonas State University, Manaus, Amazonas, Brazil; f University of Pernambuco, Recife, Pernambuco, Brazil; g Division of Cardiology, LSU Health Shreveport, Shreveport, LA 71115, USA

**Keywords:** Drug-coated balloon, Drug-eluting stent, *De novo* coronary artery disease, Regional differences, Confounding

## Abstract

**Background:**

Randomized trials of drug-coated balloons (DCBs) versus drug-eluting stents (DESs) for *de novo* coronary artery disease (dnCAD) are confined to Europe and Asia, raising the question of whether outcomes vary by region. We aimed to examine whether outcomes differ between regions and, if so, whether any difference reflects geography itself or trial-level confounding.

**Methods:**

PubMed, Embase, and Cochrane Central were searched for randomized trials comparing DCB with DES in dnCAD. Incidence rate ratios (IRRs) were pooled with a random-effects model (DerSimonian-Laird; Hartung-Knapp confidence intervals (CIs)). The pre-specified primary question was regional (Europe vs Asia) subgroup comparison of a harmonized device-oriented composite outcome (DOCO). To determine whether any regional signal reflected geography itself or trial-level confounding, we performed subgroup-aware leave-one-out analysis and examined two alternative explanations *post-hoc*: vessel-size inclusion and dominance of the single largest trial. We also descriptively examined comparator-DES generation across trials.

**Results:**

Thirteen trials (4,673 randomized patients) were included; the primary DOCO analysis comprised 12 trials (4,396 patients). DOCO did not differ overall (IRR = 1.00; 95% CI, 0.70–1.44; I^2^ = 49.1%). The pre-specified regional difference was statistically significant (Europe IRR = 0.83; 95% CI, 0.44–1.57; Asia IRR = 1.48; 95% CI, 1.04–2.11; subgroup P = 0.04) but was not robust on subgroup-aware leave-one-out analysis: the subgroup-interaction P value rose above 0.05 in eight of 12 iterations, most prominently on omission of REC-CAGEFREE I (about 49% of randomized patients; subgroup P value from 0.04 to 0.46), indicating a regional contrast driven by trial-level imbalance rather than geography. Vessel size and comparator-DES generation differed systematically between regions. No secondary endpoint differed overall, including cardiac death (IRR = 1.20; 95% CI, 0.94–1.52). All 13 trials used paclitaxel-coated balloons.

**Conclusions:**

The apparent regional difference appears largely attributable to trial-level confounding (vessel size, comparator generation, single-trial dominance) rather than to geography itself. Because the randomized evidence comes only from Europe and Asia, these findings show that the Europe–Asia difference is not robust, but do not rule out geographic effects in other regions. Because all current randomized evidence is paclitaxel-specific and confined to Europe and Asia, globally representative trials of limus-coated balloons against contemporary DES are required.

## Introduction

Drug-eluting stents (DESs) are the standard treatment for *de novo* coronary artery disease, but the permanent metallic scaffold carries a lifelong risk of in-stent restenosis and stent thrombosis. Drug-coated balloons (DCBs) deliver a lipophilic antiproliferative agent without a permanent implant, have established efficacy in in-stent restenosis, and are increasingly studied in *de novo* lesions. A recent meta-analysis reported overall equivalence between DCB and DES in this indication [1]. Because the randomized evidence is confined to European and Asian trials, the possibility of regional variation in outcomes—and whether any such pattern reflects geography itself or trial-level confounding—has not been formally examined. We conducted this meta-analysis to address that question directly.

## Materials and Methods

This systematic review followed the Preferred Reporting Items for Systematic Reviews and Meta-Analyses (PRISMA) 2020 [2] and was prospectively registered in PROSPERO (CRD420251178732). Institutional review board approval was not applicable, as this study is a secondary analysis of published aggregate data and did not involve individual patient data or new human-subjects research. This study was conducted in compliance with the ethical standards of the responsible institution on human subjects as well as with the Helsinki Declaration.

PubMed, Embase, and the Cochrane Central Register were searched from database inception to November 18, 2025, with a targeted update in February 2026 limited to follow-up reports of already-included trials, so any newly eligible trial published thereafter would not have been captured (Supplementary Material 1, cr.elmerpub.com). Randomized trials enrolling adults with *de novo* coronary artery disease, comparing DCB with DES, with at least 6 months’ follow-up and at least one outcome of interest, were eligible. Trials of in-stent restenosis, bifurcation lesions, or DCB-versus-bare-metal-stent comparisons were excluded. Two reviewers screened titles/abstracts and full texts independently; disagreements were resolved by discussion with a third reviewer.

The primary outcome was a harmonized device-oriented composite outcome (DOCO), defined per trial as major adverse cardiovascular events (MACEs), target-lesion failure (TLF), or device-oriented clinical endpoint (DoCE); trial-specific labels and component definitions are listed here (Supplementary Material 2a, cr.elmerpub.com). Secondary outcomes were target-lesion revascularization (TLR), all-cause mortality, cardiac death, myocardial infarction, and target-vessel or stent thrombosis. For trials with serial follow-up publications, the longest available follow-up reported in a full peer-reviewed publication was used; the follow-up reports merged into each parent trial are listed (Supplementary Material 3, cr.elmerpub.com).

Incidence rate ratios (IRRs) per person-year were pooled with a random-effects model (DerSimonian-Laird τ^2^; Hartung-Knapp confidence intervals (CIs)). IRR was selected as the primary metric because follow-up varied widely across trials (6 months to 5 years) and incidence rates account for differential person-time at risk; conventional risk ratios (RRs) were computed as a sensitivity analysis. The log-IRR standard error was calculated as √(1/e_1_ + 1/e_2_); a 0.5 continuity correction was applied only when a study had a zero-event cell. Estimates for sparse-event outcomes (cardiac death, vessel thrombosis) are correspondingly sensitive to this correction. Person-years were reconstructed from the average number at risk over each reported follow-up interval (Supplementary Material 2b, cr.elmerpub.com). The number of trials contributing differs by outcome because not every trial reported every endpoint. The geographic subgroup analysis was pre-specified in the registered PROSPERO protocol and was the review’s principal question. To determine whether any regional difference was robust, we performed subgroup-aware leave-one-out analysis (recomputing the regional subgroup estimates and the subgroup-interaction P value with each trial omitted in turn) and examined two alternative explanations *post-hoc*: vessel size (small-vessel versus broader inclusion) and dominance of single largest trial; comparator-DES generation (first- versus second-generation) was described qualitatively. We additionally performed post-hoc univariable random-effects meta-regression of the primary outcome on follow-up duration, vessel-size category, and comparator-DES generation; a sensitivity analysis using conventional RRs; and a sensitivity analysis restricted to trials with target-lesion-revascularization–based composite definitions. Risk of bias was assessed using Risk of Bias 2 tool (RoB 2) [3]; certainty was assessed using Grading of Recommendations Assessment: Development and Evaluation (GRADE) [4]; small-study effects were assessed using Egger’s test for the primary outcome (*k* = 12). P values were two-sided, with a significance threshold of P < 0.05. Analyses were conducted using R 4.5.2 (meta package). Additional methods, per-trial event counts and person-years, device classification, GRADE summary, and robustness analyses are provided here (Supplementary Materials 1–6, cr.elmerpub.com).

## Results

**Figure 1:**
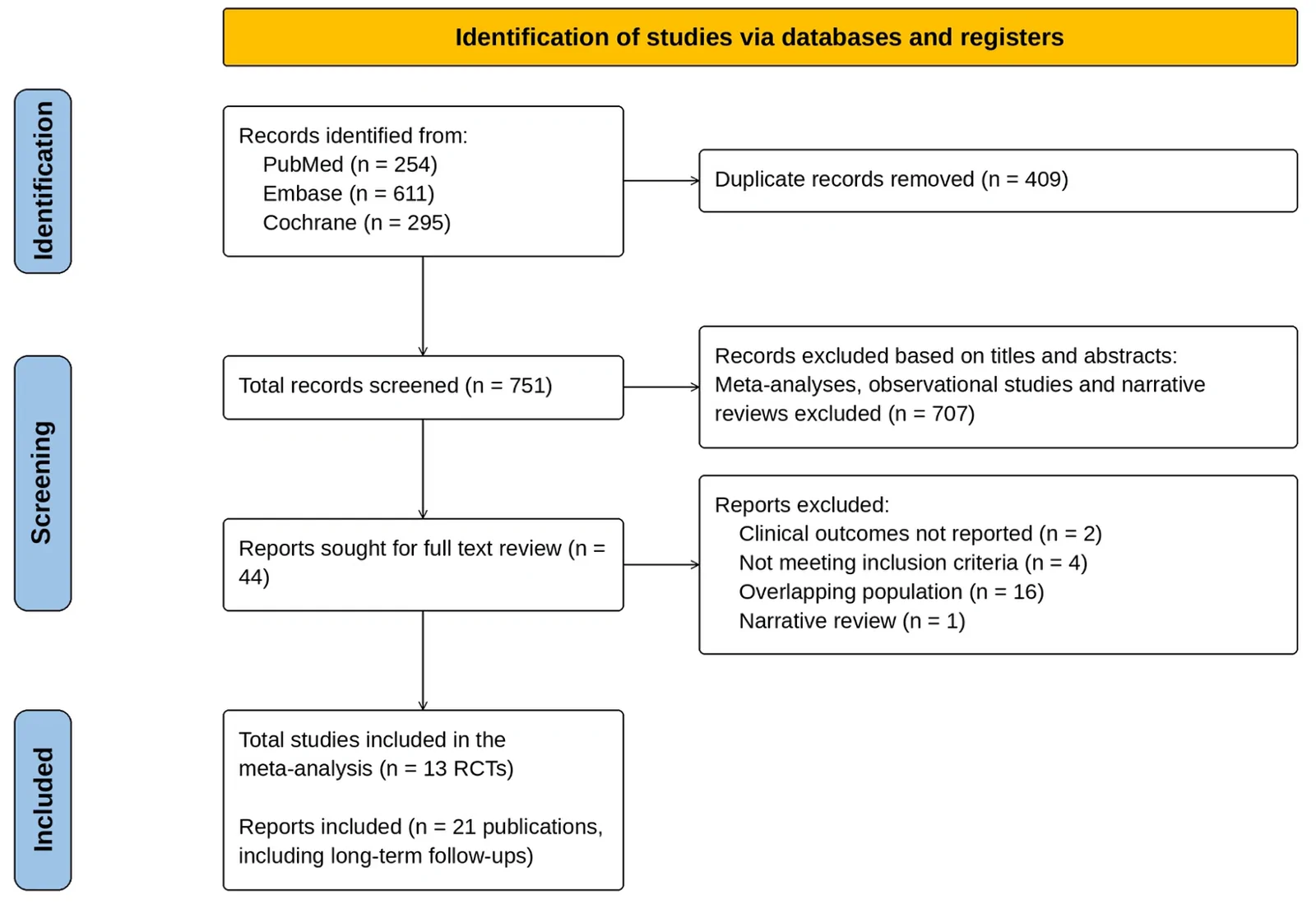
PRISMA 2020 flow diagram of study identification, screening, and inclusion. Records were identified from PubMed (n = 254), Embase (n = 611), and Cochrane Central (n = 295); after removal of 409 duplicates, 751 records were screened, of which 706 were excluded at title and abstract review. Forty-five reports underwent full-text review (clinical outcomes not reported, n = 2; inclusion criteria not met, n = 4; overlapping population, n = 16; narrative review, n = 1; excluded studies are listed (Supplementary Material 5, cr.elmerpub.com), yielding 13 randomized controlled trials reported across 21 publications. PRISMA: Preferred Reporting Items for Systematic Reviews and Meta-Analyses; RCT: randomized controlled trial.

The search identified 1,160 records; 751 were screened after de-duplication and 44 underwent full-text review, yielding 13 randomized trials reported across 21 publications (Fig. 1; Supplementary Material 5, cr.elmerpub.com) [5–17]. Across the systematic review, 4,673 patients were randomized (DCB 2,343; DES 2,330); the primary DOCO analysis included 12 trials and 4,396 patients (Nishiyama et al [17] reported only TLR). Six trials were European and seven Asian; follow-up ranged from 6 months to 5 years. All 13 trials used a paclitaxel-coated balloon. Pooled estimates for all outcomes (overall and by region) are summarized in Table 1.

**Table 1:** Pooled incidence rate ratios for DCB versus DES, overall and by region

Outcome	*k*	Events DCB/DES	Overall IRR (95% CI)	I^2^	Europe IRR (95% CI)	Asia IRR (95% CI)	Subgroup P
DOCO (primary)	12	210/190	1.00 (0.70–1.44)	49.1%	0.83 (0.44–1.57)	1.48 (1.04–2.11)	0.04
TLR	11	94/65	1.24 (0.72–2.14)	46.3%	0.94 (0.23–3.86)	2.03 (1.19–3.44)	0.12
All-cause mortality	9	89/81	1.10 (0.92–1.31)	0%	0.94 (0.60–1.48)	1.23 (1.12–1.36)	0.07
Cardiac death	11	57/47	1.20 (0.94–1.52)	0%	1.37 (0.95–1.98)	1.11 (0.71–1.75)	0.33
Myocardial infarction	12	59/75	0.80 (0.62–1.03)	0%	0.69 (0.41–1.16)	0.93 (0.73–1.17)	0.19
TV / stent thrombosis	11	6/14	0.64 (0.37–1.10)	0%	0.37 (0.13–1.02)	1.03 (0.64–1.65)	0.01

Random-effects (DerSimonian-Laird τ^2^) IRRs with Hartung-Knapp 95% CIs; IRR > 1 favors DES. *k*: number of trials contributing; DOCO: device-oriented composite outcome; TLR: target-lesion revascularization; TV: target-vessel; DCB: drug-coated balloon; DES: drug-eluting stent; CI: confidence interval; IRR: incidence rate ratio.

### Primary outcome and regional difference

**Figure 2:**
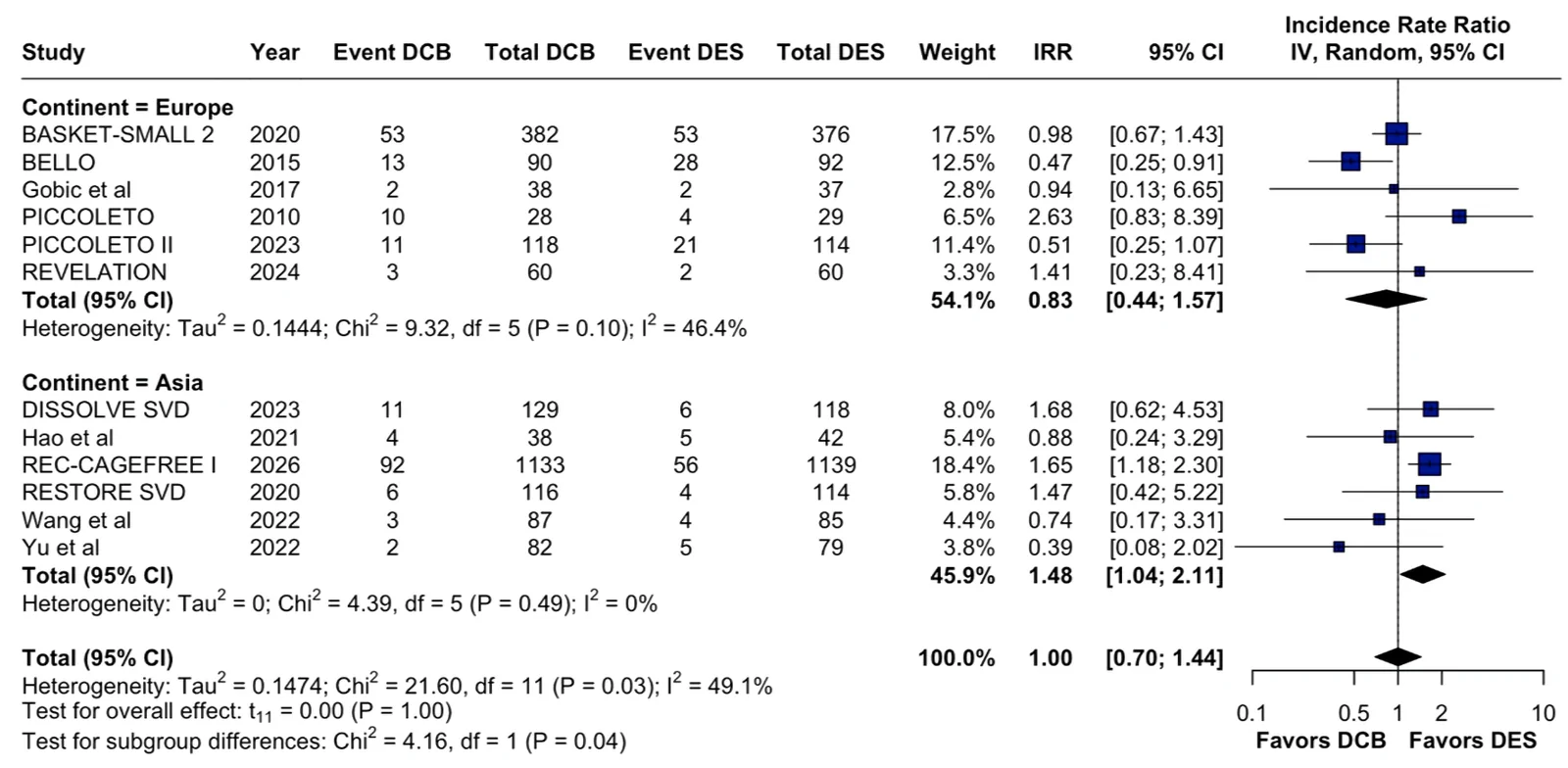
Random-effects forest plot of the device-oriented composite outcome (DOCO), with trials stratified by continent of enrollment (pre-specified subgroup, PROSPERO CRD420251178732). Overall IRR = 1.00 (95% CI, 0.70–1.44), I^2^ = 49.1%; Europe 0.83 (0.44–1.57); Asia 1.48 (1.04–2.11); test for subgroup differences χ^2^ = 4.16, df = 1, P = 0.04, *k* = 12. DCB: drug-coated balloon; DES: drug-eluting stent; CI: confidence interval; IRR: incidence rate ratio.

DOCO did not differ overall between DCB and DES (IRR = 1.00; 95% CI, 0.70–1.44; I^2^ = 49.1%). In the pre-specified regional subgroup analysis by geography, European trials showed no difference (IRR = 0.83; 95% CI, 0.44–1.57) while Asian trials favored DES (IRR = 1.48; 95% CI, 1.04–2.11), with a statistically significant subgroup difference (χ^2^ = 4.16, df = 1; P = 0.04) (Fig. 2). However, this regional signal was not robust. In subgroup-aware leave-one-out analysis, the subgroup-interaction P value rose above 0.05 in 8 of 12 iterations: three of six Asian trials (RESTORE SVD, DISSOLVE SVD, REC-CAGEFREE I) and five of six European trials (BELLO, BASKET-SMALL 2, PICCOLETO II, REVELATION, Gobic et al [10]) individually nullified the subgroup difference when omitted. The most extreme single-trial effect was observed for REC-CAGEFREE I, the largest trial (contributing about 49% of randomized patients), where omission shifted the subgroup-interaction P value from 0.04 to 0.46 (Supplementary Material 6a, b, cr.elmerpub.com). REC-CAGEFREE I nevertheless contributed only 18.4% of the random-effects weight (despite approximately 49% of randomized patients); with its removal, the pooled DOCO estimate was an IRR of 0.88 (95% CI, 0.61–1.27), and I^2^ fell from 49.1% to 21.4% (Supplementary Material 6c, cr.elmerpub.com). The breadth of this fragility indicates that the regional contrast is not a stable feature of the data but rather a consequence of trial-level imbalance. Second, vessel size differed systematically between regions: four of six European trials enrolled patients with small vessels (≤ 2.75 mm), in which DCB and DES perform similarly, compared with only two of seven Asian trials, whereas the largest Asian REC-CAGEFREE I trial enrolled patients irrespective of vessel size and found DCB inferior to DES, with the effect concentrated in larger vessels (Supplementary Material 6d, cr.elmerpub.com) [13]. Third, the comparator differed by era: early European trials largely used paclitaxel-eluting stents initially [5–7], whereas nearly all Asian trials used second-generation limus-eluting stents (Supplementary Material 4a, cr.elmerpub.com) [11–14, 16, 17]. Continent is thus collinear with vessel size, comparator generation, and one dominant trial. Univariable meta-regression identified no significant moderator of the primary outcome—follow-up duration (β = −0.008, P = 0.57), vessel-size category (β = −0.22, P = 0.54), and comparator-DES generation (β = −0.13, P = 0.74)—consistent with limited power (*k* = 11–12) and collinearity among these study-level factors and continent (Supplementary Material 4b, cr.elmerpub.com). Findings were materially unchanged using conventional RRs (RR = 1.00; 95% CI, 0.69–1.45; regional subgroup P = 0.05), and restricting the analysis to trials with target-lesion-revascularization–based composite definitions left the overall estimate null (IRR = 1.28; 95% CI, 0.82–2.00) while attenuating the regional contrast (subgroup P = 0.40) (Supplementary Material 4c, cr.elmerpub.com).

### Secondary outcomes

No secondary endpoint showed a robust overall difference (Table 1). TLR did not differ overall (IRR = 1.24; 95% CI, 0.72–2.14; I^2^ = 46.3%), although the Asian subgroup favored DES (IRR = 2.03; 95% CI, 1.19–3.44) while the European subgroup did not (subgroup difference P = 0.12). All-cause mortality did not differ (IRR = 1.10; 95% CI, 0.92–1.31). Cardiac death did not differ (IRR = 1.20; 95% CI, 0.94–1.52; I^2^ = 0%) and was directionally concordant with all-cause mortality. Myocardial infarction did not differ (IRR = 0.80; 95% CI, 0.62-1.03). Target-vessel or stent thrombosis did not differ overall (IRR = 0.64; 95% CI, 0.37–1.10); the thrombosis subgroup difference (Europe 0.37, Asia 1.03; subgroup P = 0.01) rests on very sparse events and is hypothesis-generating only.

### Risk of bias, certainty, and small-study effects

Most trials were at low overall risk of bias; a minority raised some concerns due to deviations or missing data; none were at high risk (Supplementary Material 4d, cr.elmerpub.com). By GRADE, certainty was moderate for all-cause mortality, cardiac death, myocardial infarction, and thrombosis, and low for DOCO and TLR (Supplementary Material 4e, cr.elmerpub.com). Egger’s test for the primary outcome was nonsignificant (*t* = –0.76, P = 0.46).

## Discussion

In 13 randomized trials and 4,673 patients, DCB and DES produced comparable overall outcomes for *de novo* coronary artery disease, consistent with the prior pooled estimate of O’Callaghan et al [1]. The pre-specified regional subgroup analysis by geography showed a statistically significant difference (P = 0.04). On further interrogation of this signal, the data did not support geography as a determinant of DCB-versus-DES outcomes; they showed that trial design, lesion selection, and comparator era drive the between-region appearance. The symmetry of the leave-one-out evidence should be acknowledged: the regional signal is fragile across multiple subgroup-aware iterations, and the overall null estimate is similarly sensitive to REC-CAGEFREE I, reflecting trial-level imbalance on both sides of the comparison rather than a stable regional or overall effect. Because individual patient data were unavailable, the associations with vessel size and comparator generation are ecological (study-level) and cannot establish patient-level causation; they are best interpreted as sources of confounding of the regional contrast rather than independently quantifiable effects. Antiplatelet regimens likewise differed across trials and regions—several Asian DCB-only protocols used abbreviated dual antiplatelet therapy—and, being unmeasured at the patient level, may further contribute to the between-region appearance.

A structural gap in the current evidence deserves emphasis: every randomized DCB arm to date uses paclitaxel; no completed randomized trial compares a limus-coated balloon with a contemporary DES in this indication. Pooled paclitaxel-DCB estimates should not be extrapolated to the limus devices increasingly used in practice; the TRANSFORM II and SELUTION DeNovo trials will likely provide globally representative, contemporary-comparator data [18, 19].

### Limitations

Two limitations emphasized in this review warrant particular note. First, the pooled and regional estimates were disproportionately influenced by a single trial: REC-CAGEFREE I contributed roughly half of the randomized patients, though random-effects weighting reduced its share of the pooled estimate to 18.4%. Despite this downweighing, both the overall null and the regional signal remained sensitive to its inclusion, reflecting trial-level imbalance rather than a stable effect. Second, all included trials were conducted in Europe or Asia, with no randomized data from North America, South America, Africa, or Oceania; the present findings therefore bear on the robustness of the Europe–Asia contrast and cannot exclude geographic effects globally.

Aggregate (not individual-patient) data were used, so the composite endpoint had to be harmonized across partially differing trial definitions—combining trial-defined endpoints with different components (target-vessel- versus target-lesion-based revascularization)—and confounders could not be adjusted at the patient level; the associations with vessel size, comparator-DES generation, and continent are therefore ecological and hypothesis-generating rather than a patient-level causal decomposition. Because follow-up durations differed widely (6 months to 5 years), outcomes were pooled as incidence-rate ratios using person-years reconstructed from the average numbers at risk under a within-interval constant-hazard assumption, which could introduce bias if event rates varied over time. Univariable meta-regression was underpowered (*k* = 11–12) and, given the collinearity of continent, vessel size, and comparator generation, could not isolate their independent contributions. Finally, antiplatelet regimens differed across trials and regions—several Asian DCB-only protocols used abbreviated dual antiplatelet therapy—and, being unmeasured at the patient level, may further contribute to the between-region appearance.

### Conclusions

The apparent Europe–Asia difference between DCB and DES for *de novo* coronary disease appears largely attributable to trial-level confounding—vessel size, comparator-DES generation, and dominance of a single large trial—rather than to geography itself. Because the randomized evidence comes only from Europe and Asia, these findings show that the Europe–Asia difference is not robust, but do not rule out geographic effects in other regions. Current randomized evidence remains paclitaxel-specific and confined to Europe and Asia. Globally representative trials of contemporary DCB platforms against current-generation DES, with balanced lesion selection, are required before any regional or class-level conclusions can be drawn.

## Supplementary Material

Suppl 1 Database search strategies.

Suppl 2 Endpoint definitions and per-trial outcome data.

Suppl 3 Long-term follow-up reports merged into parent trials.

Suppl 4 Trial-level covariates, analyses, and quality assessment.

Suppl 5 Studies excluded at full-text review, with reasons (n = 23).

Suppl 6 Robustness and subgroup analyses of the primary outcome.

## Data Availability

All extracted data are available from the corresponding author on reasonable request.

## Abbreviations

ACS: acute coronary syndrome
CCS: chronic coronary syndrome
CI: confidence interval
DAPT: dual antiplatelet therapy
DCB: drug-coated balloon
DES: drug-eluting stent
dnCAD: *de novo* coronary artery disease
DOCO: device-oriented composite outcome
DoCE: device-oriented clinical endpoint
GRADE: Grading of Recommendations Assessment, Development and Evaluation
IRR: incidence rate ratio
ISR: in-stent restenosis
MACE: major adverse cardiovascular events
MI: myocardial infarction
PCI: percutaneous coronary intervention
PRISMA: Preferred Reporting Items for Systematic Reviews and Meta-Analyses
PROSPERO: International Prospective Register of Systematic Reviews
RCT: randomized controlled trial
RoB 2: Risk of Bias 2 tool
TLF: target-lesion failure
TLR: target-lesion revascularization
TVR: target-vessel revascularization
