# Supplementary material for: Regional Differences Between Drug-Coated Balloons and Drug-Eluting Stents in De Novo Coronary Disease
Source: Cardiol Res. 2026 Aug 31;17(5):545–51. doi: 10.14740/cr2252 (PMC13568751; doi:10.14740/cr2252)
Supplement: Supplementary file 1 [file cr-17-05-545-s001.docx]

**Suppl 1.** Database search strategies

**PubMed**

("drug-coated balloon" OR DCB OR "drug-eluting balloon" OR DEB OR "paclitaxel-coated balloon" OR PCB OR "paclitaxel-eluting balloon" OR PEB OR "sirolimus-coated balloon" OR SCB OR "sirolimus-eluting balloon" OR SEB) AND ("drug-eluting stent" OR DES) AND ("coronary artery disease" OR "myocardial infarction" OR "percutaneous coronary intervention" OR PCI OR "de novo")

**Embase**

('drug-coated balloon' OR DCB OR 'drug-eluting balloon' OR DEB OR 'paclitaxel-coated balloon' OR PCB OR 'paclitaxel-eluting balloon' OR PEB OR 'sirolimus-coated balloon' OR SCB OR 'sirolimus-eluting balloon' OR SEB) AND ('drug-eluting stent' OR DES) AND ('coronary artery disease' OR 'myocardial infarction' OR 'percutaneous coronary intervention' OR PCI OR 'de novo')

**Cochrane Central Register of Controlled Trials**

Same Boolean structure as PubMed, with double-quoted phrases.
