# Supplementary material for: Regional Differences Between Drug-Coated Balloons and Drug-Eluting Stents in De Novo Coronary Disease
Source: Cardiol Res. 2026 Aug 31;17(5):545–51. doi: 10.14740/cr2252 (PMC13568751; doi:10.14740/cr2252)
Supplement: Supplementary file 2 [file cr-17-05-545-s002.docx]

**Suppl 2.** Endpoint definitions and per-trial outcome data

**Suppl 2a.** Composite-endpoint label and components by trial (harmonized for the primary outcome)

| **Trial** | **Label** | **Components** |
| --- | --- | --- |
| PICCOLETO | MACE | Death, new ST-elevation MI, and TLR |
| BELLO | MACE | Death, Q- or non-Q-wave MI, or TVR |
| BASKET-SMALL 2 | MACE | Cardiac death, non-fatal MI, and TVR |
| PICCOLETO II | TLF | Cardiac death, all MI, TLR |
| REVELATION | MACE | Cardiac death, recurrent MI, TLR |
| Gobić et al | MACE | Cardiac death, recurrent MI, TLR |
| RESTORE SVD | TLF | Cardiac death, target-vessel MI, ischemia-driven TLR |
| DISSOLVE SVD | TLF | Cardiac death, target-vessel MI, ischemia-driven TLR |
| REC-CAGEFREE I | DoCE | Cardiovascular death, target-vessel MI, clinically and physiologically indicated TLR |
| Yu et al | MACE | Cardiac death, non-fatal MI, TLR, TVR |
| Hao et al | MACE | Cardiac death, recurrent MI, TLR |
| Wang et al | MACE | Cardiac death, non-fatal MI, TVR, and stent thrombosis |
| Nishiyama et al | MACE | Not reported (TLR-only data extracted) |

**Suppl 2b.** Per-trial events and person-years for the primary outcome (DOCO)

*Per-trial denominators, events, and person-years contributing to the primary device-oriented composite outcome analysis (k = 12 trials, 4,396 patients).*

| **Trial** | **FU (mo)** | **Analyzed DCB/DES** | **Events DCB** | **Events DES** | **Person-years DCB** | **Person-years DES** |
| --- | --- | --- | --- | --- | --- | --- |
| PICCOLETO | 9 | 28/ 29 | 10 | 4 | 21.4 | 22.5 |
| BELLO | 36 | 83 / 83 | 13 | 28 | 264.0 | 269.3 |
| BASKET-SMALL 2 | 36 | 349 / 345 | 53 | 53 | 1,095.0 | 1,071.5 |
| RESTORE SVD | 24 | 115 / 109 | 6 | 4 | 229.5 | 225.5 |
| PICCOLETO II | 36 | 102 / 101 | 11 | 21 | 323.0 | 317.0 |
| DISSOLVE SVD | 12 | 129 / 118 | 11 | 6 | 129.0 | 118.0 |
| REC-CAGEFREE I | 36 | 1,104 / 1,106 | 92 | 56 | 3,375.5 | 3,388.5 |
| Yu et al | 12 | 82 / 79 | 2 | 5 | 83.5 | 82.0 |
| REVELATION | 60 | 56 / 51 | 3 | 2 | 283.5 | 265.6 |
| Gobić et al | 6 | 38 / 37 | 2 | 2 | 19.8 | 18.5 |
| Hao et al | 12 | 38 / 42 | 4 | 5 | 38.0 | 42.0 |
| Wang et al | 12 | 87 / 85 | 3 | 4 | 89.5 | 88.5 |

*Person-years computed as (mean number at risk over each follow-up interval) × interval length, summed across all reported intervals (constant-hazard assumption within intervals). Nishiyama et al did not report DOCO and contributed only to the target-lesion revascularization analysis (k = 11; pooled estimate in main Table 1).*
