# Supplementary material for: Regional Differences Between Drug-Coated Balloons and Drug-Eluting Stents in De Novo Coronary Disease
Source: Cardiol Res. 2026 Aug 31;17(5):545–51. doi: 10.14740/cr2252 (PMC13568751; doi:10.14740/cr2252)
Supplement: Supplementary file 3 [file cr-17-05-545-s003.docx]

**Suppl 3.** Long-term follow-up reports merged into parent trials

Thirteen randomized trials were reported across 21 publications; where a trial had serial follow-up reports, the longest available follow-up reported in a full peer-reviewed publication was used and all reports were treated as a single trial (not double-counted). The follow-up reports merged into their parent trials were:

- BELLO — Latib 2012 (6 mo), Naganuma 2015 (24 mo), Latib 2015 (36 mo; used).
- BASKET-SMALL 2 — Jeger 2018 (12 mo), Jeger 2020 (36 mo; used).
- RESTORE SVD — Tang 2018 (12 mo), Tian 2020 (24 mo; used for DOCO; 3-year outcomes reported only as a conference abstract [TCT Connect 2020], not used).
- PICCOLETO II — Cortese 2020 (12 mo), Cortese 2023 (36 mo; used).
- REC-CAGEFREE I — Gao 2024 (24 mo), Tao 2026 (36 mo; used).
- REVELATION — Vos 2019 (9 mo), Niehe 2022 (24 mo), Niehe 2024 (60 mo; used).
