# Supplementary material for: Regional Differences Between Drug-Coated Balloons and Drug-Eluting Stents in De Novo Coronary Disease
Source: Cardiol Res. 2026 Aug 31;17(5):545–51. doi: 10.14740/cr2252 (PMC13568751; doi:10.14740/cr2252)
Supplement: Supplementary file 4 [file cr-17-05-545-s004.docx]

**Suppl 4.** Trial-level covariates, analyses, and quality assessment

**Suppl 4a.** DCB and DES by trial region

| **Trial** | **Continent** | **DCB device (manufacturer)** | **DES device** |
| --- | --- | --- | --- |
| PICCOLETO | Europe | Dior PCB (Eurocor, Germany) | Taxus Liberté |
| BELLO | Europe | IN.PACT Falcon (Medtronic Invatec, Italy) | Taxus Liberté |
| BASKET-SMALL 2 | Europe | SeQuent Please (B. Braun, Germany) | Taxus Element / Xience |
| PICCOLETO II | Europe | Elutax SV (Aachen Resonance, Germany) | Xience (everolimus) |
| REVELATION | Europe | Pantera Lux (Biotronik, Germany) | Orsiro / Xience |
| Gobić et al | Europe | SeQuent Please (B. Braun, Germany) | BioMime (sirolimus) |
| RESTORE SVD | Asia | SafePAX Restore (Cardionovum, Germany) | Resolute Integrity |
| DISSOLVE SVD | Asia | Dissolve (DK Medical, China) | Endeavor Resolute |
| REC-CAGEFREE I | Asia | Swide (Shenqi Medical, China) | Firebird 2 (sirolimus) |
| Yu et al | Asia | SeQuent Please (B. Braun, Germany) | Mixed 2nd-gen DES |
| Hao et al | Asia | Yinyi Bingo (Liaoning, China) | Not reported |
| Wang et al | Asia | Vasoguard (Rientech, China) | Cordimax (sirolimus) |
| Nishiyama et al | Asia | SeQuent Please (B. Braun, Germany) | Xience Prime / Xpedition |

**Suppl 4b.** Univariable random-effects meta-regression of the primary outcome (DOCO)

| **Moderator** | **k** | **β (log-IRR)** | **SE** | **p** |
| --- | --- | --- | --- | --- |
| Follow-up duration (per month) | 12 | −0.008 | 0.013 | 0.57 |
| Vessel-size category (small vs broader) | 12 | −0.223 | 0.347 | 0.54 |
| Comparator-DES generation (1st- vs 2nd-gen) | 11 | −0.134 | 0.388 | 0.74 |

*Random-effects meta-regression (DerSimonian–Laird τ²) with Knapp–Hartung variance adjustment; test statistic on t distribution with k–2 df. No moderator was statistically significant. Hao et al did not report the comparator-DES device and was excluded from the DES-generation model (k=11). The non-significance is consistent with limited power (11–12 trials) and collinearity among continent, vessel size, and comparator generation; these study-level factors cannot be disentangled with aggregate data.*

**Suppl 4c.** Sensitivity analyses for the primary outcome (DOCO): risk ratios and endpoint harmonization

| **Analysis** | **k** | **Overall (95% CI)** | **I²** | **Europe (95% CI)** | **Asia (95% CI)** | **Subgroup p** |
| --- | --- | --- | --- | --- | --- | --- |
| Primary (IRR) | 12 | 1.00 (0.70–1.44) | 49.1% | 0.83 (0.44–1.57) | 1.48 (1.04–2.11) | 0.04 |
| Risk ratio (RR) | 12 | 1.00 (0.69–1.45) | 56.0% | 0.85 (0.44–1.65) | 1.48 (1.04–2.11) | 0.05 |
| TLR-based composites only (IRR) | 8 | 1.28 (0.82–2.00) | 30.5% | 1.06 (0.31–3.67) | 1.59 (1.23–2.05) | 0.40 |

*Risk ratios (inverse-variance, DerSimonian–Laird τ², Hartung–Knapp) reproduced the IRR findings: the overall estimate was null and the regional contrast was again borderline and non-robust. The endpoint-harmonization analysis restricted the primary outcome to the eight trials whose composite used target-lesion (not target-vessel) revascularization (excluding BELLO, BASKET-SMALL 2, Yu et al, and Wang et al, whose composites included TVR); the overall estimate remained null and the regional difference attenuated (p=0.40).*

**Suppl 4d.** RoB 2 domain judgements

| **Trial** | **D1** | **D2** | **D3** | **D4** | **D5** | **Overall** |
| --- | --- | --- | --- | --- | --- | --- |
| PICCOLETO | Low | Some concerns | Low | Low | Low | Some concerns |
| BELLO | Low | Low | Low | Low | Low | Low |
| BASKET-SMALL 2 | Low | Some concerns | Low | Low | Low | Low |
| PICCOLETO II | Low | Low | Low | Low | Low | Low |
| REVELATION | Low | Low | Some concerns | Some concerns | Low | Some concerns |
| Gobić et al | Some concerns | Some concerns | Low | Low | Low | Some concerns |
| RESTORE SVD | Low | Low | Low | Low | Low | Low |
| DISSOLVE SVD | Low | Low | Low | Low | Low | Low |
| REC-CAGEFREE I | Low | Low | Low | Low | Low | Low |
| Yu et al | Low | Low | Low | Low | Low | Low |
| Hao et al | Some concerns | Some concerns | Low | Low | Low | Some concerns |
| Wang et al | Low | Low | Low | Low | Low | Low |
| Nishiyama et al | Some concerns | Some concerns | Low | Low | Low | Some concerns |

*D1 = randomization process; D2 = deviations from intended interventions; D3 = missing outcome data; D4 = measurement of the outcome; D5 = selection of the reported result. Overall judgement per RoB 2 algorithm.*

**Suppl 4e.** GRADE certainty of evidence for DCB versus DES, by outcome and geographic subgroup

*Certainty was rated using the GRADE approach across the five core domains (risk of bias, inconsistency, indirectness, imprecision, and publication bias) and is reported overall and by geographic subgroup (Europe vs Asia) for each outcome included in the meta-analysis. Crude incidences (events / analyzed N) are shown for context and are not the pooled effect estimates; pooled incidence rate ratios with 95% confidence intervals are reported in Table 1 of the main manuscript.*

| **Region** | **No. of trials** | **Quality assessment** | | | | | | **Crude incidences** | | **Overall certainty** |
| --- | --- | --- | --- | --- | --- | --- | --- | --- | --- | --- |
|  |  | **Study design** | **Risk of bias** | **Inconsistency** | **Indirectness** | **Imprecision** | **Publication bias** | **DCB (events/N)** | **DES (events/N)** |  |
| **Device-oriented composite outcome (DOCO)** | | | | | | | | | | |
| **Europe** | 6 | RCT | Not serious | Serious | Not serious | Serious | Not serious | 92/712 | 110/712 | **Low** |
| **Asia** | 6 | RCT | Not serious | Not serious | Serious | Not serious | Not serious | 118/1575 | 80/1566 | **Moderate** |
| **Overall** | 12 | RCT | Not serious | Serious | Not serious | Serious | Not serious | 210/2287 | 190/2278 | **Low** |
| **Target-lesion revascularization (TLR)** | | | | | | | | | | |
| **Europe** | 4 | RCT | Not serious | Serious | Not serious | Very serious | Not serious | 26/292 | 31/299 | **Very low** |
| **Asia** | 7 | RCT | Not serious | Not serious | Serious | Not serious | Not serious | 102/3201 | 34/1599 | **Moderate** |
| **Overall** | 11 | RCT | Not serious | Serious | Not serious | Serious | Not serious | 128/3493 | 65/1898 | **Low** |
| **All-cause mortality** | | | | | | | | | | |
| **Europe** | 4 | RCT | Not serious | Not serious | Not serious | Serious | Not serious | 35/614 | 37/615 | **Moderate** |
| **Asia** | 5 | RCT | Not serious | Not serious | Serious | Not serious | Not serious | 53/1493 | 43/1487 | **High** |
| **Overall** | 9 | RCT | Not serious | Not serious | Not serious | Serious | Not serious | 88/2107 | 80/2102 | **Moderate** |
| **Cardiac death** | | | | | | | | | | |
| **Europe** | 6 | RCT | Not serious | Not serious | Not serious | Serious | Not serious | 21/712 | 14/712 | **Moderate** |
| **Asia** | 5 | RCT | Not serious | Not serious | Serious | Not serious | Not serious | 36/1493 | 33/1487 | **High** |
| **Overall** | 11 | RCT | Not serious | Not serious | Not serious | Not serious | Not serious | 57/2205 | 47/2199 | **High** |
| **Myocardial infarction** | | | | | | | | | | |
| **Europe** | 6 | RCT | Not serious | Not serious | Not serious | Serious | Not serious | 28/712 | 41/712 | **Moderate** |
| **Asia** | 6 | RCT | Not serious | Not serious | Serious | Serious | Not serious | 31/1575 | 34/1566 | **Low** |
| **Overall** | 12 | RCT | Not serious | Not serious | Not serious | Serious | Not serious | 59/2287 | 75/2278 | **Moderate** |
| **Target-vessel or stent thrombosis** | | | | | | | | | | |
| **Europe** | 5 | RCT | Not serious | Not serious | Not serious | Serious | Not serious | 2/674 | 10/675 | **Moderate** |
| **Asia** | 6 | RCT | Not serious | Not serious | Serious | Serious | Not serious | 4/1575 | 4/1566 | **Moderate** |
| **Overall** | 11 | RCT | Not serious | Not serious | Not serious | Serious | Not serious | 6/2249 | 14/2241 | **Moderate** |

*GRADE certainty levels:* ***High*** *— we are very confident that the true effect lies close to the estimate;* ***Moderate*** *— moderately confident; the true effect is likely close to the estimate, but possibly substantially different;* ***Low*** *— confidence is limited; the true effect may differ substantially from the estimate;* ***Very low*** *— very little confidence; the true effect is likely substantially different.*

*Downgrading rationale: inconsistency was rated serious for DOCO and TLR overall and in Europe because of moderate between-trial heterogeneity (I² 46–49%); indirectness was rated serious for Asian subgroups owing to enrolment restricted to Asian populations; imprecision was rated serious for outcomes with wide confidence intervals crossing the null and very serious for European TLR (sparse events with very wide CI); risk of bias was not serious overall, with no trials at high overall risk on RoB 2; publication bias was not serious for the primary outcome (Egger's test t = −0.76, p = 0.46).*

*DCB, drug-coated balloon; DES, drug-eluting stent; DOCO, device-oriented composite outcome; GRADE, Grading of Recommendations Assessment, Development and Evaluation; RCT, randomized controlled trial; TLR, target-lesion revascularization. The number of trials contributing differs by outcome because not every trial reported every endpoint.*
