# Supplementary material for: Regional Differences Between Drug-Coated Balloons and Drug-Eluting Stents in De Novo Coronary Disease
Source: Cardiol Res. 2026 Aug 31;17(5):545–51. doi: 10.14740/cr2252 (PMC13568751; doi:10.14740/cr2252)
Supplement: Supplementary file 5 [file cr-17-05-545-s005.docx]

**Suppl 5.** Studies excluded at full-text review, with reasons (n = 23)

*Records that could appear to meet the inclusion criteria but were excluded at full-text assessment (PRISMA 2020, item 16b). Counts by reason match the Figure 1 flow diagram: overlapping population, n = 16; inclusion criteria not met, n = 4; clinical outcomes not reported, n = 2; narrative review, n = 1.*

| **#** | **Author, year** | **Journal** | **Reason for exclusion** |
| --- | --- | --- | --- |
| 1 | Wang and Gao, 2025 | JACC | overlapping population, Poster - sub-group analysis of REC-CAGEFREE I for impact of sex on outcomes |
| 2 | Song, 2025 | JACC | overlapping population, Poster - sub-group analysis of REC-CAGEFREE I for impact of LVEF on outcomes |
| 3 | Lv, 2025 | JACC | Poster. Overlapping population - sub-group analysis of REC-CAGEFREE I; for diffuse CAD |
| 4 | Li, 2025 | JACC | Poster. Overlapping population - sub-group analysis of REC-CAGEFREE I; for coronary bifurcation |
| 5 | Leone and Colombo, 2025 | European Heart Journal | Review/Summary of REC-CAGEFREE I |
| 6 | Cortese, 2025 | JACC | Poster - No clinical outcome reported; TRANSFORM II BASIC trial |
| 7 | Cattaneo, 2025 | J Am Heart Assoc | Overlapping population of BASKET-SMALL 2 - evaluating cost-effectiveness |
| 8 | Baghdasaryan, 2025 | JACC | Wrong population. Evaluating Complex coronary lesions. Poster of PREVAIL DCB study ; adequate data unavailable for statistical analysis |
| 9 | Rubini Gimenez, 2024 | Clin Res Cardiol | Overlapping population of BASKET-SMALL 2 - evaluating sex-specific inequalities subgroups |
| 10 | Seeger, 2023 | J Cardiovasc Dev Dis | Overlapping population of BASKET-SMALL 2 - evaluating impact of insulin on diabetes subgroups |
| 11 | Ke, 2023 | Rev Cardiovasc Med | Wrong population. Evaluating True or complex coronary bifurcation lesions |
| 12 | Scheller, 2022 | Circ Cardiovasc Interv | Overlapping population of BASKET-SMALL 2 - evaluating high bleeding risk subgroups |
| 13 | Mangner, 2022 | Circ Cardiovasc Interv | Overlapping population of BASKET-SMALL 2 - evaluating impact of clinical presentation subgroups |
| 14 | Kawai, 2022 | Eurointervention | Trial evaluating coronary vasomotion, clincal outcome events not reported |
| 15 | Farah, 2022 | Postepy Kardiol Interwencyjnej | Overlapping population of BASKET-SMALL 2 - evaluating coronary vessel size subgroups |
| 16 | Farah, 2022 | JACC | Poster. Overlapping population of BASKET-SMALL 2 - evaluating coronary vessel size subgroups |
| 17 | Wöhrle, 2021 | JACC Cardiovasc Interv | Overlapping population of BASKET-SMALL 2 - evaluating impact of diabetes subgroups |
| 18 | Li, 2021 | Int J Cardiol | Overlapping population. RESTORE trial. |
| 19 | Scheller, 2020 | EuroIntervention | Wrong control group. PEPCAD NSTEMI trial evaluating BMS and DES vs DCB. |
| 20 | Fahrni, 2020 | Clin Res Cardiol | Overlapping population of BASKET-SMALL 2 - angiographic analysis subgroup |
| 21 | Giannini, 2017 | Cardiovasc Revasc Med | Overlapping population of BELLO trial - diabetic and non-diabetic subgroup |
| 22 | Giannini, 2013 | JACC | Poster. Overlapping population of BELLO trial - diabetic and non-diabetic subgroup |
| 23 | Poerner, 2014 | Circ Cardiovasc Interv | BMS postdilated with the paclitaxel DEB was compared with everolimus drug-eluting stents |

*Abbreviations: BASKET-SMALL 2, BELLO, REC-CAGEFREE I, and RESTORE denote the parent trials of which the excluded records were overlapping subgroup, cost, angiographic, or mechanistic reports. Posters/abstracts are indicated where applicable. Full bibliographic citations to be finalized in the journal reference format.*
