# Supplementary material for: Regional Differences Between Drug-Coated Balloons and Drug-Eluting Stents in De Novo Coronary Disease
Source: Cardiol Res. 2026 Aug 31;17(5):545–51. doi: 10.14740/cr2252 (PMC13568751; doi:10.14740/cr2252)
Supplement: Supplementary file 6 [file cr-17-05-545-s006.docx]

**Suppl 3.** Long-term follow-up reports merged into parent trials

Thirteen randomized trials were reported across 21 publications; where a trial had serial follow-up reports, the longest available follow-up reported in a full peer-reviewed publication was used and all reports were treated as a single trial (not double-counted). The follow-up reports merged into their parent trials were:

- BELLO — Latib 2012 (6 mo), Naganuma 2015 (24 mo), Latib 2015 (36 mo; used).
- BASKET-SMALL 2 — Jeger 2018 (12 mo), Jeger 2020 (36 mo; used).
- RESTORE SVD — Tang 2018 (12 mo), Tian 2020 (24 mo; used for DOCO; 3-year outcomes reported only as a conference abstract [TCT Connect 2020], not used).
- PICCOLETO II — Cortese 2020 (12 mo), Cortese 2023 (36 mo; used).
- REC-CAGEFREE I — Gao 2024 (24 mo), Tao 2026 (36 mo; used).
- REVELATION — Vos 2019 (9 mo), Niehe 2022 (24 mo), Niehe 2024 (60 mo; used).

**Suppl 6.** Robustness and subgroup analyses of the primary outcome

**Suppl 6a.** Subgroup-aware leave-one-out for the regional DOCO comparison

**Caption.** Each row shows the recomputed Asian and European pooled IRRs and the subgroup-difference p with the indicated trial omitted. Baseline subgroup p = 0.04. Omission of REC-CAGEFREE I shifted the Asian estimate from 1.48 (1.04-2.11) toward the null with subgroup p rising to 0.46, demonstrating that the regional signal is not robust to the single largest trial.

**Suppl 6b.** Leave-one-out analysis for the primary outcome (DOCO)

**Caption.** Pooled IRR for DOCO after omitting each trial in turn (random effects; Hartung-Knapp). Omitting REC-CAGEFREE I yields IRR 0.88 (95% CI 0.61-1.27), with I² falling from 49.1% to 21.4%; all other single-trial omissions leave the pooled estimate non-significant.

**Suppl 6c.** Device-oriented composite outcome (DOCO) excluding REC-CAGEFREE I

*Random-effects forest plot of DOCO after removing REC-CAGEFREE I (k=11). REC-CAGEFREE I contributed 18.4% of the random-effects weight in the primary analysis (≈49% of randomized patients); with it removed, the pooled IRR was 0.88 (95% CI 0.61–1.27) and I² fell from 49.1% to 21.4%, and the Europe–Asia subgroup difference became non-significant (p=0.46).*

**Suppl 6d.** DOCO stratified by vessel-size inclusion criteria

**Caption.** Small-vessel subgroup (6 trials, upper bound ≤2.75 mm): IRR 0.94 (95% CI 0.48-1.85; I² 56.4%). Broader-vessel subgroup (6 trials): IRR 1.45 (95% CI 0.99-2.11; I² 0%). Subgroup-difference p = 0.15. Vessel-size inclusion is collinear with continent: four of six European trials enrolled small vessels versus two of seven Asian trials.
